# Interfacial Engineering of MoS_2_ Thin Films for Wettability-Dependent Resistive Switching and Neuromorphic Behaviors

**DOI:** 10.3390/nano16150959

**Published:** 2026-08-04

**Authors:** Yuhang Yang, Yuan Yu, Cancan Cui, Xin Liu, Yanyong Li, Peisong Liu, Fei Hui

**Affiliations:** 1 School of Materials Science and Engineering, Zhengzhou University, Science Avenue 100, Zhengzhou 450001, China; yyh1130@gs.zzu.edu.cn (Y.Y.);; 2 Engineering Research Center for Nanomaterials (ERCN), National & Local Joint Engineering Research Center for Applied Technology, Henan University, Zhengzhou 450001, China; 3 Key Laboratory of Materials Physics, Ministry of Education, School of Physics, Zhengzhou University, Science Avenue 100, Zhengzhou 450001, China

**Keywords:** MoS_2_, interfacial engineering, large-area preparation, memristors, synaptic plasticity

## Abstract

Recent years have witnessed a surge in the research of memristors as fundamental building blocks for neuromorphic computing, owing to their exceptional ability to emulate the plastic behavior of biological synapses in a high-density, low-power hardware format. These devices are increasingly recognized as the key to achieving efficient artificial neural networks. Two-dimensional (2D) molybdenum disulfide (MoS_2_) is a premier candidate for artificial synapses due to its atomic scale and tunable electronic properties. However, achieving wafer-scale MoS_2_ thin films for integrated memristor systems remains a significant challenge. In this work, a scalable strategy combining cetyltrimethylammonium bromide (CTAB)-assisted electrochemical intercalation and oil–water interface self-assembly was developed to fabricate large-area 2H-phase MoS_2_ thin films. Leveraging the amphiphilic nature of CTAB-functionalized MoS_2_ nanosheets, continuous Janus-structured MoS_2_ films with asymmetric wetting properties (hydrophilic vs. hydrophobic) were successfully prepared. Vertical-structured Ag/Janus-structured MoS_2_/ITO memristors demonstrated robust non-volatile switching with high endurance and long-term retention. The devices successfully emulated biological synaptic behaviors, including short-term and long-term plasticity. Furthermore, the memristors exhibited distinct optoelectronic synergistic modulation under 405 nm illumination, enabling light-sensitive synaptic functions. This work offers a versatile interface engineering route for low-power integrated sensing–memory–computing hardware.

## 1. Introduction

The rapid proliferation of data-intensive technologies, such as artificial intelligence and big data analytics, has exposed the inherent limitations of the traditional von Neumann architecture [1]. The physical separation of memory and processing units leads to significant energy dissipation and latency during high-frequency data shuttling—a phenomenon known as the “von Neumann bottleneck” [2]. As the device dimensions continue to scale down, the continuation of traditional Moore’s Law is confronting fundamental physical limits; moreover, solely relying on size reduction fails to resolve the inherent inefficiency stemming from the separation of memory and processing units [3,4,5]. Consequently, neuromorphic computing, inspired by the low-power consumption and massively parallel processing of the biological brain, has emerged as a promising non-von Neumann alternative [6,7,8,9]. Among various candidates, memristors are regarded as ideal hardware building blocks for emulating biological synapses due to their non-volatile switching, rapid operation, and energy efficiency [10,11,12]. However, traditional memristors employing metal-oxide functional layers are inherently limited by the stochasticity arising from the random migration of oxygen vacancies, which restricts their application in high-density integrated circuits [13,14]. Consequently, exploring emerging functional materials and designing memristors with enhanced performance have become pivotal research directions.

The emergence of low-dimensional materials, spearheaded by the discovery of graphene [15], has illuminated novel directions for memristor research and generated widespread attention within the academic community [16,17]. Unlike conventional bulk materials, two-dimensional (2D) materials possess atomic-scale thickness and an exceptionally high surface-to-volume ratio. Their outstanding mechanical flexibility also renders them ideal candidates for flexible and wearable electronic [18,19]. Furthermore, the exceptional sensitivity of 2D materials to external electric fields facilitates precise control over ionic migration and charge transport [20,21,22,23]. This capability not only reduces the switching energy but also effectively suppresses stochastic fluctuations, thereby paving a new way for the development of highly stable, low-power memristive devices for artificial synaptic applications [19,20,21]. Among various 2D materials, transition metal dichalcogenides (TMDs) [24] stand out for their diverse band structures and superior electrical properties. In particular, as a typical TMD member, molybdenum disulfide (MoS_2_) possesses a tunable direct bandgap [25] and outstanding photoresponse, demonstrating immense potential for applications in optoelectronic and memory devices [22,26,27,28,29].

Xiong et al. constructed TiN/O–MoS_2_ memristors using mechanical exfoliation, effectively reducing the operation voltage and significantly enhancing the switching ratio through oxygen-doping engineering [30], while Ranganathan et al. employed an in situ “post-sulfurization” chemical vapor deposition (CVD) strategy to grow MoS_2_ thin films for memristor fabrication, achieving long-term stability in both high and low resistance states to boost non-volatile memory performance [31]. Furthermore, Paolo Samorì et al. utilized supramolecular engineering to construct synaptic device based on 2D asymmetrically functionalized MoS_2_ film, achieving independent regulation of optoelectronic dual-modes and the flexible switching from short-term plasticity (STP) to long-term plasticity (LTP) [32]. These studies demonstrate that the vertical layered structure of MoS_2_ provides natural van der Waals gap channels, which greatly facilitates the migration of active metal ions. In fact, defect engineering, particularly the precise generation, regulation, and fabrication of atomic sulfur vacancies (V_s_) within TMDs, has been extensively recognized as a pivotal strategy to tailor local electronic structures and optimize resistive switching (RS) characteristics [33,34]. With the continuous advancement of technology, exploring large-area fabrication of high-quality MoS_2_ thin films has become indispensable for achieving the arrayed integration of high-performance MoS_2_ memristors. While traditional mechanical exfoliation can produce MoS_2_ nanosheets with superior intrinsic properties, its low yield and substantial variability among exfoliated flakes impede large scale manufacturing [30]. Liquid-phase exfoliation features facile processing, low cost, and process compatibility, yet it has shortcomings in terms of large-area uniformity and film continuity [35,36]. Although CVD is a promising route for wafer-scale continuous MoS_2_ films, its high growth temperatures poses significant integration challenges with silicon-based processes or flexible substrates [37,38]. Therefore, developing a MoS_2_ thin-film fabrication technology that balances large-scale preparation and processing flexibility holds significant practical importance. However, during the transfer and integration of large-area thin films, precisely regulating the interfacial characteristics between 2D materials and substrates, as well as suppressing charge scattering and the disordered growth of conductive filaments, remains a major challenge. In particular, there is still a lack of systematic research on how interfacial molecular alignment affects the physicochemical properties of thin films, thereby intervening in or even reshaping the underlying RS mechanisms of memristors. To address these issues, this paper proposes a scalable fabrication scheme based on CTAB-assisted electrochemical intercalation and oil–water interfacial self-assembly [39,40], successfully constructing a continuous Janus-structured MoS_2_ thin film with asymmetric polarity (hydrophilic on one side and hydrophobic on the other). This study systematically elucidates the regulation principles of the interfacial molecular modification layer on the ion migration process, providing a novel theoretical foundation for the uniformity control and mechanism design of high-performance memristor arrays.

In this work, CTAB was employed as an electrochemical intercalant to exfoliate natural bulk MoS_2_ crystals. Benefiting from its inherent steric hindrance, high-quality, few-layer MoS_2_ nanosheets with favorable dispersibility were successfully prepared. Using a phase-interface transfer strategy, CTAB-functionalized MoS_2_ nanosheets were self-assembled at the oil/water interfaces to form Janus-structured MoS_2_ thin films with distinct hydrophilic and hydrophobic surfaces. On the basis, vertical-structure memristors (Ag/Janus-structured MoS_2_/ITO) were then constructed to evaluate the RS behaviors governed by the different interfaces. Mechanistic exploration demonstrates that the organic modification layer on the hydrophobic side serves as a crucial ion barrier, switching the dominant RS mechanism from the conventional electrochemical metallization (ECM) on the hydrophilic side to a highly controllable intrinsic valence change mechanism (VCM) dominated by V_s_. Furthermore, through systematic pulse characterization, the device exhibits a successful transition from short-term to long-term plasticity and nearly linear long-term potentiation/depression (LTP/LTD) characteristics. Additionally, leveraging its nonlinear optoelectronic synergistic modulation under programmed light stimuli, the device achieved an MNIST recognition accuracy exceeding 90%. These quantitative metrics demonstrate superior synaptic functional stability, establishing a robust experimental foundation for Janus-structured MoS_2_ film memristors in high-efficiency neuromorphic hardware.

## 2. Materials and Methods

### 2.1. Chemicals

All chemical reagents were of analytical grade and utilized without additional purification. N,N-dimethylformamide (DMF) (99%, Aladdin Reagent Co., Shanghai, China), Cetyltrimethylammonium bromide (CTAB) (99%, Aladdin Reagent Co., Shanghai, China), Acetonitrile (Chengdu Kelong Chemical Co., Chengdu, China), Polyvinylpyrrolidone (PVP) (K12, Shanghai Macklin Biochemical Co., Shanghai, China), Absolute ethanol (Tianjin Miou Chemical Reagent Co., Shanghai, China), Petroleum ether (Sinopharm Chemical Reagent Co., Shanghai, China), n-Hexane (Shanghai Titan Scientific Co., Shanghai, China), Chloroform (Aladdin Reagent Co., Shanghai, China).

### 2.2. Preparation of MoS_2_ Nanosheet Dispersion

A natural MoS_2_ crystal block (width × length × thickness: 1 cm × 1 cm × 1 mm) was employed as the cathode and fixed in the electrochemical cell using a pure copper alligator clip, while a high-purity graphite rod served as the anode. The electrolyte consisted of 100 mL of 5 mg/mL CTAB solution in acetonitrile. A constant DC voltage of 8 V was applied to drive the intercalation proceed for 3 h. Upon completion, the expanded MoS_2_ was rapidly transferred into a mixture of 40 mL DMF and 0.4 g PVP, immediately followed by bath sonication for 1 h. The resulting suspension was purified via sequential centrifugation: initially at 3000 rpm for 3 min to remove unexfoliated bulk MoS_2_, after which the supernatant was collected and further centrifuged at 10,000 rpm for 15 min to harvest the nanosheet pellet. Finally, the obtained pellet was washed three times with isopropanol (10,000 rpm, 15 min per cycle) and redispersed in 30 mL anhydrous isopropanol to form a stable MoS_2_ nanosheet dispersion.

### 2.3. Transfer of Janus MoS_2_ Film from the Oil–Water Interface

A stable liquid–liquid interface was established in a 60 mL glass vessel to facilitate the self-assembly of MoS_2_ nanosheets. To configure an upward-facing hydrophobic surface, an oil/water (O/W) interface was formed by successive addition of 20 mL ultrapure water and 20 mL n-hexane (or petroleum ether). In contrast, for transferring films with a hydrophilic surface, a water/chloroform system was established by sequentially adding 20 mL of chloroform and 20 mL of ultrapure water. The prepared MoS_2_ nanosheet dispersion was extracted using a micropipette, and the tip was inserted into the liquid–liquid interface. The dispersion was slowly injected at the interface, accompanied by mild shaking to promote the spread and assembled into a continuous, large-area thin film. In this study, this asymmetrically functionalized material defined as “Janus-structured MoS_2_ film”, where distinct chemical properties (i.e., hydrophobic and hydrophilic) are achieved on opposite surfaces via interfacial self-assembly.

### 2.4. Substrate Pretreatment and Device Construction

The cleaned silicon wafers and ITO conductive glass were placed face-up and subjected to oxygen plasma treatment using a plasma cleaner to render their surfaces super-hydrophilic. Silicon wafers were treated at 70 W for 600 s, while the ITO conductive glass was processed at 35 W for 600 s. For flexible ITO substrates, the plasma treatment was conducted at 35 W for 360 s. Specifically, plasma-treated substrates were employed for transferring Janus-structured MoS_2_ films with the hydrophobic side facing upward. Conversely, standard-cleaned substrates (without plasma treatment) were utilized to transfer Janus-structured MoS_2_ films with the hydrophilic side facing upward.

Metal silver (Ag) was deposited as the top electrode on the surface of the transferred Janus-structured MoS_2_ thin films by thermal evaporation through a metal shadow mask. The deposition process was conducted under a vacuum level larger than 7 × 10^−4^ Pa, reaching a final thickness of 80 nm. The top electrodes were defined by the mask as a rectangular array of dots with a diameter of 100 μm, while the pre-treated ITO conductive layer served directly as the bottom electrode. The resulting Ag/MoS_2_/ITO vertical sandwich structure devices were stored in a nitrogen-filled glovebox prior to testing to minimize the impact of environmental moisture and oxygen on the interfaces.

### 2.5. Characterization

The microstructure of the as-prepared MoS_2_ was analyzed using transmission electron microscopy (TEM; model JEOL JEM2100 plus, JEOL Ltd., Akishima, Tokyo, Japan). The morphology of MoS_2_ were analyzed by field emission scanning electron microscopy (SEM; model Supra55, Carl Zeiss AG, Oberkochen, Germany). The Raman spectra were collected by an HR800 Raman microscope (Horiba Jobin Yvon, Longjumeau, France), and XPS spectra were collected by X-ray photoelectron spectroscopy (XPS; model Nexsa, Thermo Fisher Scientific, Waltham, MA, USA). Attenuated total reflection Fourier transform infrared spectroscopy (ATR-FTIR) was recorded on a Bruker Vertex-70 spectrophotometer (Bruker Corporation, Billerica, MA, USA). The water contact angle was recorded with a DM300 liquid–solid interface analyzer (Kyowa Interface Science Co., Ltd., Saitama, Japan). Optical microscope pictures were collected with a Leica DM4000M (Leica Microsystems GmbH, Wetzlar, Germany). Topographic AFM maps were collected using a JPK NanoWizard AFM (JPK Instruments AG, Berlin, Germany) in tapping mode, provided with Si tips (model NCH from Nanoworld AG, Neuchâtel, Switzerland).

## 3. Results and Discussion

### 3.1. Preparation of MoS_2_ Dispersions and Thin Films

**Figure 1:**
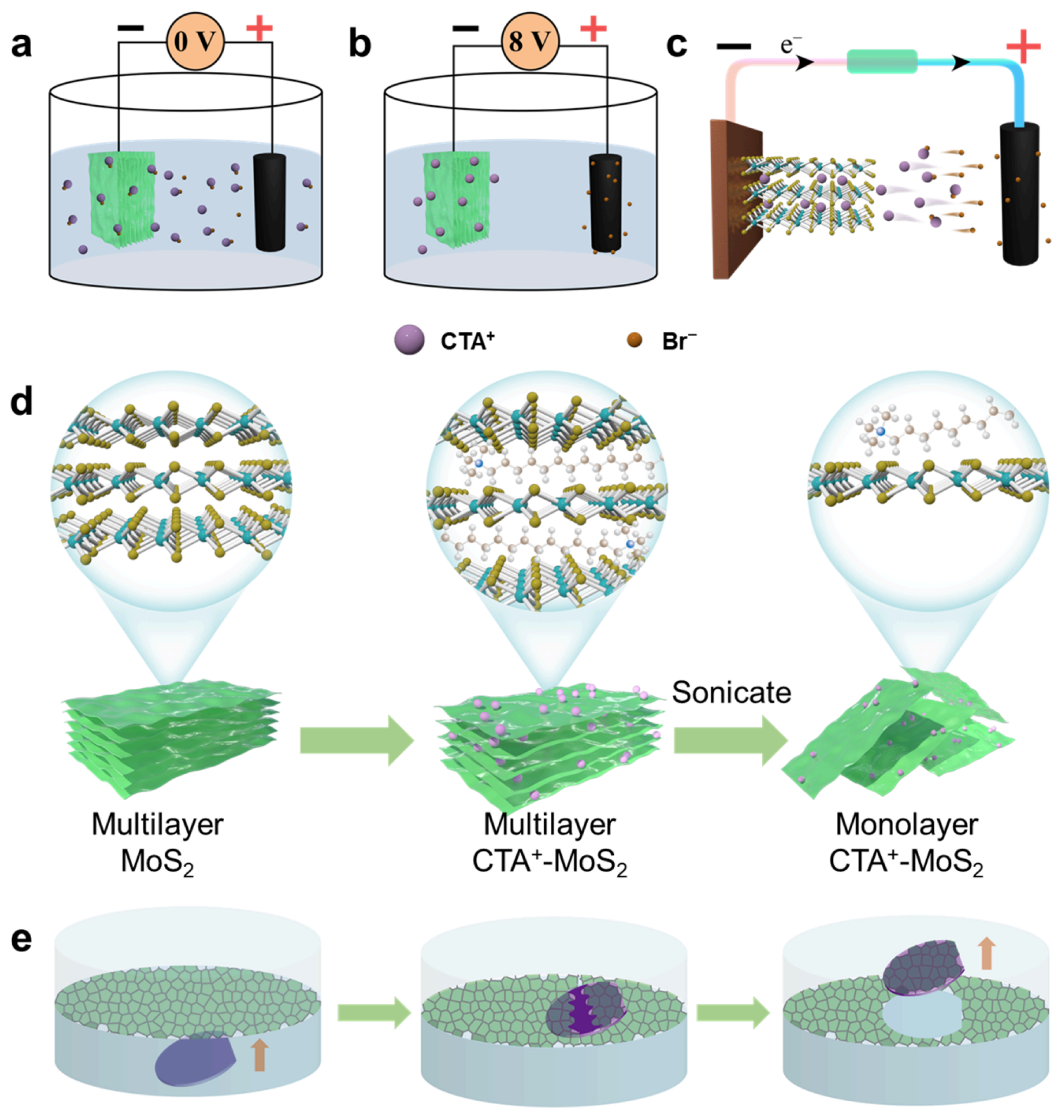
Schematic illustration of the preparation process for 2H-phase MoS_2_ nanosheet dispersion and the oil–water interfacial film formation. (**a**–**c**) Schematic of the electrochemical intercalation reaction between MoS_2_ and CTAB. (**a**) CTAB uniformly dispersed in acetonitrile without an applied voltage. (**b**) Upon applying an 8 V voltage, Br^−^ migrates toward the anode while CTA^+^ moves toward the cathode. (**c**) Driven by the electric field, CTA^+^ overcomes the interlayer energy barrier and embeds into the vdWs gaps of MoS_2_, forming an interlayer intercalation compound. The arrow represents the direction of electron flow (e−). (**d**) The insertion of bulky CTA^+^ ions significantly expands the interlayer spacing of MoS_2_, effectively weakening the interlayer vdWs coupling. Subsequently, through liquid-phase ultrasonication, the expanded bulk structure undergoes cleavage and is exfoliated into few-layer MoS_2_ nanosheets. (**e**) MoS_2_ nanosheets are drop-cast at the oil–water interface for self-assembly into a film. A clean silicon substrate is then slowly lifted from below (indicated by the upward orange arrow), allowing the continuous MoS_2_ film to be uniformly transferred onto its surface. The horizontal green arrows indicate the sequence of the transfer process.

In this work, an electrochemical reaction cell was constructed using a natural bulk MoS_2_ crystal as the cathode and a graphite rod as the anode. Without an applied voltage, CTAB serves as the intercalant molecule and is uniformly dispersed in the acetonitrile electrolyte (Figure 1a). Upon applying a steady voltage of 8 V, the reaction is activated (Figure 1b). Driven by the electric field, the negatively charged bromide ions (Br^−^) migrate toward the graphite anode, where a yellow substance appears (Figure S1a) due to anodic oxidation. Simultaneously, the MoS_2_ cathode undergoes significant morphological changes. As intercalation progresses, the insertion of cetyltrimethylammonium cations (CTA^+^) gradually expands the interlayer spacing of the MoS_2_ layered structure (Figure 1c). After 3 h, the MoS_2_ crystal, originally characterized by distinct layers and a metallic luster, undergoes violent volume expansion. The layers spread open, exhibiting a fluffy appearance and a dark gray color; the thickness increases from 1.5 mm to approximately 11 mm, representing a tenfold macroscopic volume expansion (Figure S1b). At this stage, a large amount of Br^−^ is precipitated, turning the entire electrolyte pale yellow (Figure S1c). This significant expansion confirms the successful insertion of CTA^+^ into the MoS_2_ vdWs gaps, effectively weakening the interlayer vdWs forces. The MoS_2_ crystals expanded through the intercalation reaction shown in Figure 1a–c were transferred into a DMF mixture solution containing PVP for ultrasonication (Figure 1d).

Afterwards, a series of qualitative characterizations were conducted. ATR-FTIR (Figure S2a) reveals broad high-frequency peaks in the 2870 to 2903 cm^−1^ region and a sharp peak at 2832 cm^−1^, which correspond to the symmetric and antisymmetric stretching vibrations of C-H bonds in the CTAB alkyl chains. This confirms the selective adsorption of surfactant molecules at the interface. Under ultrasonication, the “expanded” layered structure easily disintegrates, exfoliating from multilayer structures into few-layer or even single-layer nanosheets, which are then dispersed in anhydrous isopropanol to form a stable MoS_2_ nanosheet dispersion. The as-prepared MoS_2_ dispersion appears dark green, distinct from the black color characteristic of the 1T phase. This color is a macroscopic indicator of a high-concentration, few-layer 2H-phase MoS_2_ dispersion. Raman spectroscopy (Figure S2b) shows that the $E_{2g}^{1}$ and A_1g_ peaks are perfectly preserved without the appearance of 1T metallic phase characteristic peaks (J_1_, J_2_, J_3_), indicating that the bulky organic cations (CTA^+^) do not trigger a 2H-to-1T phase transition during intercalation. UV-Vis absorption spectra (Figure S2c) exhibit three characteristic fingerprint peaks, consistent with the dark green appearance. A strong and relatively sharp primary absorption peak at 434 nm serves as a clear optical signature of successfully obtained semiconducting nanosheets. In contrast, 1T-phase dispersions typically appear black and exhibit featureless, smooth absorption curves due to their metallic nature. TEM characterization (Figure S3) shows electron diffraction patterns identical to those of the natural crystal, displaying six-fold symmetry and clear (100) and (110) diffraction spots, further confirming the preservation of the original 2H semiconducting phase. Additionally, SEM and AFM images (Figures S4 and S5) confirm the successful exfoliation into ultrathin, few-layer structures.

After successfully exploring the fundamental process of electrochemical intercalation and exfoliation for MoS_2_ nanosheets, a systematic investigation of various key parameters was conducted (Figures S6–S8) to obtain a MoS_2_ nanosheet dispersion with uniform thickness, large lateral dimensions, and suitability for oil–water interfacial film assembly. The optimal experimental protocol for preparing the MoS_2_ nanosheet dispersion was determined as follows: utilizing a 5 mg/mL CTAB/acetonitrile solution as the electrolyte, performing intercalation at a voltage of 8 V for 3 h, followed by ultrasonic exfoliation in a DMF solution containing PVP for 1 h, and finally purifying the crude dispersion by centrifugation at a speed of 3000 rpm. To achieve high-quality continuous films, an interfacial self-assembly and dip-coating transfer technique was employed (Figure 1e). The MoS_2_ dispersion was added to an immiscible oil/water interface (e.g., petroleum ether/water). In this process, the interfacial tension drives the MoS_2_ nanosheets to spontaneously arrange and pack closely at the interface, forming a dense film. The target substrate was then placed in the lower aqueous phase and slowly pulled vertically upward to transfer the MoS_2_ film onto the substrate surface (Figure S9). After solvent evaporation, a large-area, uniform MoS_2_ thin film was obtained, providing a robust material foundation for subsequent device fabrication and performance testing.

### 3.2. Morphology and Structure Characterization of MoS_2_ Thin Films

**Figure 2:**
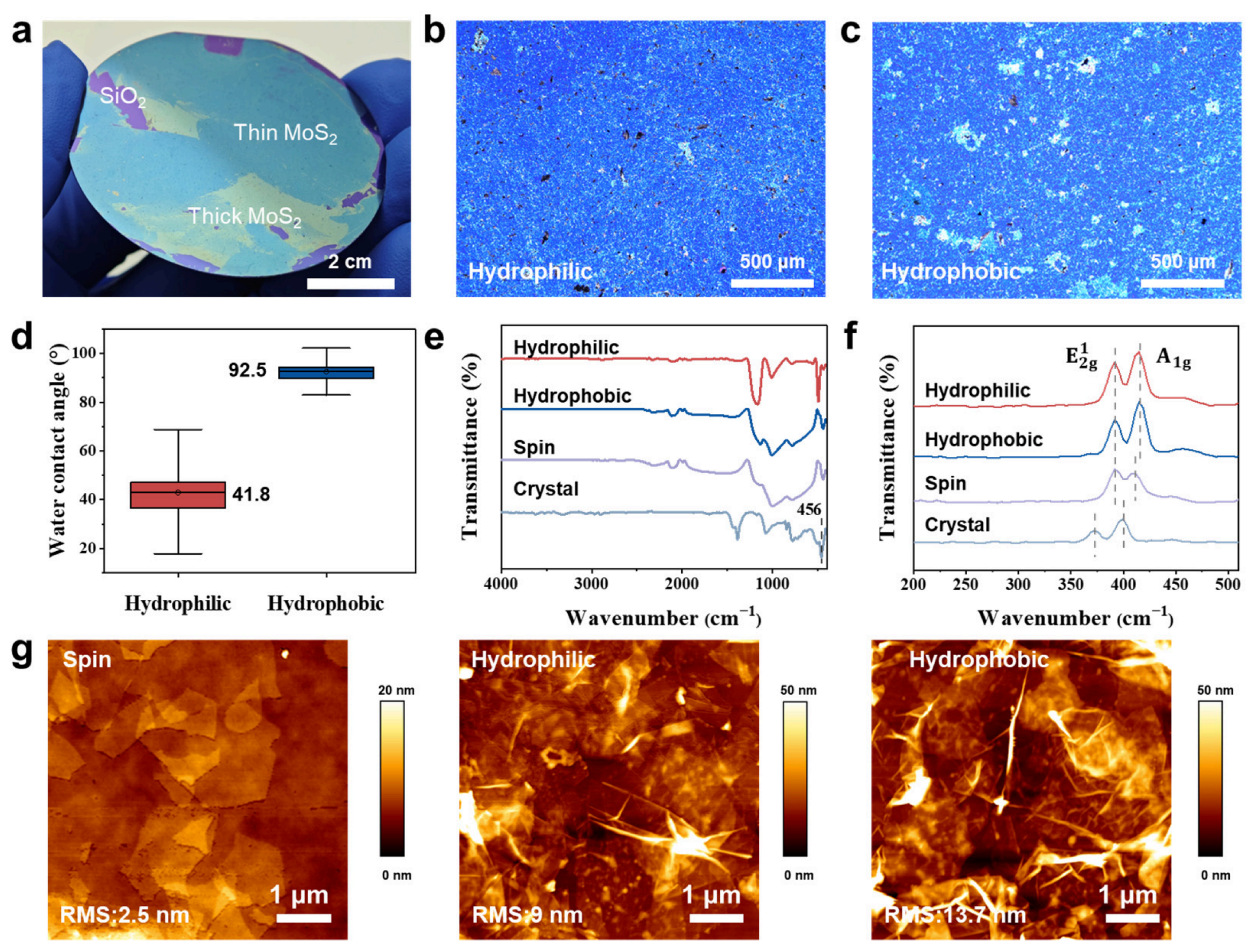
Morphological and structural characterization of MoS_2_ films. (**a**) Photograph of a MoS_2_ film transferred onto a 2-inch silicon wafer. The distinct colors (purple, blue, and light cyan) correspond to SiO_2_ substrate, thin and thick MoS_2_ films, respectively. (**b**,**c**) Optical images of the hydrophilic and hydrophobic surface of the MoS_2_ film attached to a silicon substrate; scale bar: 500 µm. (**d**) Contact angle comparison of the hydrophilic and hydrophobic sides of the CTAB-MoS_2_ film. (**e**,**f**) FT-IR and Raman spectra of drop-cast MoS_2_ nanosheet dispersion, spin-coated MoS_2_ film, and the hydrophilic/hydrophobic sides of the MoS_2_ film, respectively. The dashed line and the number “456” indicate the characteristic vibration peak at 456 cm^−1^, which corresponds to the Mo–S bond. (**g**) AFM morphology of the spin-coated MoS_2_ nanosheet dispersion and the hydrophilic/hydrophobic surfaces.

Large-area MoS_2_ thin films were successfully transferred onto 2-inch silicon wafers via an interfacial assembly approach (Figure 2a). This demonstrates the scalability and continuity of the proposed process for wafer-scale film formation. To evaluate the quality of the MoS_2_ film, a series of characterization analyses were performed on films transferred from different oil–water interfaces. Optical microscopy comparison of the hydrophilic side and hydrophobic side of the film transferred onto a SiO_2_/Si substrate (Figure 2b,c) revealed that the hydrophilic side exhibited higher flatness and smoothness, while the hydrophobic side showed a certain roughness due to the arrangement of the intercalant molecules. To quantitatively evaluate the macroscopic uniformity and reliability of the Janus characteristics, statistical water contact angle measurements were systematically performed across multiple random locations on various fabricated samples (Figure 2d). The results demonstrated distinct and highly reproducible wettability: the hydrophilic surface exhibited a mean contact angle of 41.8°, whereas the hydrophobic surface showed a mean contact angle of 92.5°, confirming the uniform coverage of the asymmetric molecular layers. This marked polarity asymmetry originates from the oriented arrangement of organic molecules with different hydrophilic/hydrophobic terminals (i.e., Janus structure) at the oil–water interface during the assembly process. The Fourier-transform infrared (FT-IR) spectrum of the pristine natural MoS_2_ crystal (Figure 2e) exhibits a characteristic peak around 456 cm^−1^, which corresponds to the characteristic vibration mode of the Mo-S bond within the MoS_2_ lattice. Infrared spectroscopy reveals that the characteristic functional group signals of MoS_2_ are preserved in both the hydrophilic and hydrophobic surface films. The emergence of broad vibrational bands (506 cm^−1^–1270 cm^−1^) in the spectra of the hydrophobic side and the spin-coated film confirmed the successful adsorption of hexadecyl chains from the CTA^+^ intercalant onto the nanosheets, forming an organic modification layer. Energy-dispersive X-ray spectroscopy (EDS, Figure S10) mapping of carbon distribution showed a lower C content on the hydrophilic side (42%) than on the hydrophobic side (55%). In this work, CTAB serves as an ultra-thin interfacial driving agent to guide the assembly of these Janus-structured MoS_2_ films. Unlike a bulk continuous layer, CTAB molecules form a conformal adlayer on the nanosheets, as evidenced by the uniform spatial distribution of XPS elemental signals (Figure S11). The hydrophobic side exhibits a significantly higher C content (48.76%) than the hydrophilic side (31.61%), confirming the outward-facing hydrophobic alkyl tails. Conversely, the elevated N concentration on the hydrophilic surface indicates the outward orientation of the ammonium head groups. These quantitative results provide conclusive evidence for the successful engineering of the asymmetric Janus interface, which serves as the physical foundation for the observed memristive behavior. Furthermore, AFM step-height profiles reveal that the self-assembled Janus-structured MoS_2_ films maintain a relatively consistent and uniform average thickness of approximately 20 nm (Figure S13). This multilayered architecture provides a robust barrier against electrical shorting while remaining sufficiently thin to facilitate efficient ion migration and sulfur vacancy (V_S_) generation for analog conductance modulation. UV-Vis characterization of the hydrophilic and hydrophobic sides (Figure S14) revealed that the absorption peak of the hydrophilic side underwent a small shift relative to that of the hydrophobic side, attributable to differences in the surface coverage of organic molecules.

In the Raman spectra (Figure 2f), the characteristic $E_{2g}^{1}$ and A_1g_ peaks of the pristine MoS_2_ crystal are located at 372 cm^−1^ and 399 cm^−1^, respectively. For both the spin-coated and interface-assembled Janus-structured MoS_2_ films, the $E_{2g}^{1}$ peak shifts to 391 cm^−1^, and the reduction in their separation further confirmed the few-layer nature of the exfoliated MoS_2_ nanosheets. Compared with the spin-coated film (where the A_1g_ peak is at 410 cm^−1^), the MoS_2_ film transferred from the oil–water interface showed a similar $E_{2g}^{1}$ peak position but a pronounced further blue shift in the A_1g_ peak to 414 cm^−1^, indicating that the self-assembly process at the interface promoted the formation of a densely restacked structure within the 2D plane. AFM was employed to quantitatively analyze the microscopic flatness of the films (Figure 2g). Compared to spin-coated films with poor nanosheet continuity, the hydrophilic and hydrophobic surface films obtained through interfacial assembly exhibit a more densely packed stacking structure. The increased surface roughness (RMS) originates from the overlapping and wrinkling of MoS_2_ nanosheets during interfacial self-assembly, which generates abundant edge sites and local electric-field concentration regions. These localized geometric features play a pivotal role in inducing local electric-field enhancement, thereby facilitating the deterministic generation of conducting filaments for memristive switching. It is observed that the hydrophobic surface of the Janus-structured MoS_2_ film exhibits relatively higher roughness and greater microscale inhomogeneity compared to the hydrophilic counterpart. This distinct morphological difference is inherently tied to the structural asymmetry of the Janus films. Specifically, during the interfacial self-assembly and transfer process, the long hydrophobic alkyl chains of the CTAB molecules preferentially orient upward on the hydrophobic side. This molecular-level capping layer naturally introduces additional topographic fluctuations and microscopic inhomogeneity when compared to the pristine, relatively smooth MoS_2_ basal plane on the hydrophilic side. These structural features facilitate conductive filament nucleation and ion migration, thereby reducing the switching voltage and improving the analog conductance modulation capability.

### 3.3. Resistive Switching Behaviors of Janus-Structured MoS_2_-Based Memristors

**Figure 3:**
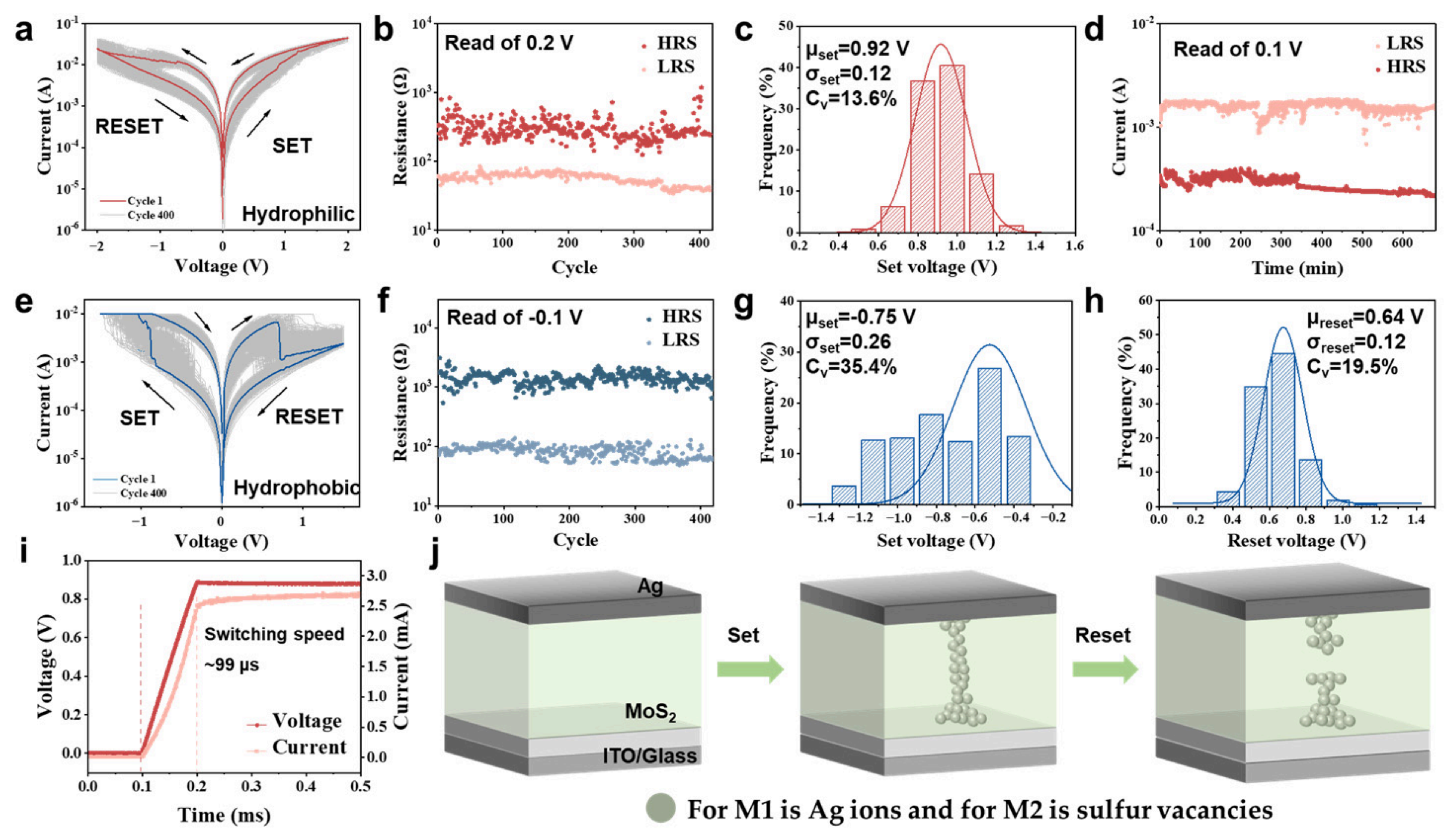
Resistive switching behaviors of Janus-structured MoS_2_ film-based devices. (**a**) I–V characteristics of the memristor based on the hydrophilic side of MoS_2_. The arrows indicate the voltage sweeping directions (0 V → +2 V → −2 V → 0 V for SET/RESET processes), and the shaded gray regions represent the distribution range of multiple consecutive I–V switching cycles. (**b**) DC cycling of the hydrophilic-side memristor. The HRS and LRS currents extracted from 400 consecutive I–V sweeps are plotted, with a read voltage of 0.1 V. (**c**) V_set_ and V_reset_ of the MoS_2_ hydrophilic surface over 400 I–V cycles were statistically analyzed. Frequency distribution histograms were plotted and fitted using a Gaussian distribution. (**d**) Data retention test of the hydrophilic-side memristor. (**e**) I–V characteristics of the memristor based on the hydrophobic side of MoS_2_. (**f**) DC cycling of the hydrophobic- surface memristor. The HRS and LRS currents from 400 I–V sweeps are shown, read at 0.1 V. (**g**,**h**) V_set_ and V_reset_ of the MoS_2_ hydrophobic surface over 400 I–V cycles were statistically analyzed. Frequency distribution histograms were plotted and fitted using a Gaussian distribution. (**i**) Switching speed of the hydrophilic-side memristor. The red dashed line indicates the onset of the voltage rise, and the light red dashed line indicates the time when the current reaches its maximum value. (**j**) Schematic illustration of the switching mechanisms for M1 based on Ag filaments and M2 based on sulfur vacancies.

Based on high-quality, continuous large-area Janus-structured MoS_2_ films, we constructed memristors using Ag as the top electrode via vacuum thermal evaporation (Figure S14). The devices with hydrophilic side facing up (Ag/Janus-structured MoS_2_/ITO, M1) exhibited typical bipolar RS behavior (Figure 3a) with highly symmetric and stable current-voltage (I–V) characteristics. During the forward sweep from 0 V to 2 V, with a compliance current of 0.1 A applied to protect the device from permanent breakdown, the device gradually transitioned from a high-resistance state (HRS) to a low-resistance state (LRS), corresponding to typical SET process. As the voltage sweeps from 2 V back to 0 V, the device current gradually decreases. A negative voltage is applied for the device to successfully switch from the LRS back to the HRS, a process corresponding to typical RESET operation of the device. Through more than 400 consecutive DC voltage sweep cycles (Figure 3b), we recorded the HRS and LRS currents at a read voltage of 0.2 V, which showed a distinct ON/OFF ratio (>10) and stable trend. This indicates that the conductive pathway, once formed, maintained good stability and repeatability under repeated electrical stressing. As shown in Figure 3a,b, with the increasing number of cycles, the resistance of the HRS exhibits a gradual trend of approaching the LRS, leading to a progressive narrowing of the resistance window between the HRS and LRS. This phenomenon is attributed to the repeated formation of conductive filaments during SET/RESET cycling, making them increasingly resistant to rupture by negative voltages, thereby causing a decrease in HRS resistance. The SET voltage (V_set_) and RESET voltage (V_reset_) extracted from the cycles were used to plot frequency distribution histograms, which were fitted with Gaussian functions (Figure 3c and Figure S16). The statistical data exhibited a clear central tendency with narrow peak widths, confirming the stable electrical performance of the device during switching cycles. Additionally, data retention tests (Figure 3d) showed that the device maintained a distinguishable on/off ratio even after 40,000 s, fully demonstrating its reliability as a non-volatile memory. Applying a 1 V voltage pulse to the hydrophilic-side memristor revealed a fast response with a turn-on speed of 99 µs (Figure 3i). The gradual current increase/decrease and rapid response indicates the device’s strong potential for emulating biological synapses in neuromorphic computing.

When the hydrophobic side faced up toward the top electrode, the fabricated memristive devices (Ag/Janus-structured MoS_2_/ITO, M2) also exhibited significant RS characteristics (Figure 3e). Cyclic DC voltage sweeps from −1.5 V to +1.5 V (under the same compliance current of 0.1 A) showed that the performance of the M2 device differed from that of the M1 device. The SET process occurred in the negative voltage sweep region (around −0.75 V), the current increased abruptly with a nearly vertical jump, indicating a transition from HRS to LRS. This sharp switching behavior is consistent with the rapid formation of conductive channels dominated by V_s_. When a positive voltage was subsequently applied, the current dropped abruptly around +0.64 V, corresponding to the RESET process where the device returned from LRS to HRS, analogous to the rupture of the sulfur vacancy filaments under the reverse electric field. The device stably cycled over 400 times within the defined voltage range. Although the SET and RESET voltages exhibited some fluctuations similar to those observed in the M1 device, the I–V window between HRS and LRS remained clearly distinguishable. The HRS and LRS currents at −0.1 V were recorded, and the corresponding resistances were calculated to plot resistance distribution histograms (Figure 3f). It is noteworthy that the M1 device exhibits a gradual resistance window narrowing due to residual species accumulation, while the M2 device maintains superior cyclic stability without detectable degradation, validating that the hydrophobic interface endows the device with outstanding robustness. To further quantify the parameter uniformity, we statistically analyzed the SET and RESET voltages during cycling and plotted their frequency distribution histograms (Figure 3g,h). Gaussian fitting revealed that both SET and RESET voltages were relatively concentrated and exhibited a degree of symmetry, demonstrating the reliability of M2 memristors for low-power operation and logic control. To evaluate the device-to-device uniformity and reproducibility, the HRS and LRS resistance distributions were statistically analyzed from 50 randomly selected memristors for both configurations (Figure S17). The tested devices exhibited stable resistive switching behaviors, corresponding to a device yield of >90%. Compared with the hydrophilic devices, the hydrophobic devices exhibit a higher average ON/OFF ratio and a tighter ON/OFF ratio distribution, demonstrating the potential of the liquid–liquid interfacial self-assembly strategy for achieving excellent the macroscopic uniformity and reliability in wafer-scale fabricated memristor devices. Furthermore, to evaluate the competitiveness of our approach, Figure S18 presents a benchmarking comparison of the endurance and retention performance of our device with representative MoS_2_-based memristors reported in the literature. The Ag/Janus-structured MoS_2_/ITO device developed in this work exhibits a balanced combination of endurance and retention, demonstrating competitive switching reliability.

The RS behavior of the devices is significantly influenced by the interface orientation (Figure 3j). For the device with the hydrophilic side facing up, the switching primarily originates from the conventional ECM mechanism. Under an applied voltage, Ag^+^ ions migrate from the top electrode under the driving electric field, leading to the formation and rupture of conductive filaments. In specific, when a positive voltage is applied, Ag^+^ ions migrate from the top electrode and are reduced at the bottom electrode. With increasing voltage, Ag accumulates at the bottom, eventually forming a conductive pathway that switches the device from HRS to LRS. Under a reverse voltage, the conductive filament ruptures due to the reverse electric field, returning the device from LRS to HRS. Notably, the hydrophobic-side film possesses an organic modification layer that acts as a buffer during RS. This organic layer effectively blocks the massive injection of Ag^+^ ions from the top electrode, shifting the dominant switching mode from extrinsic metal migration to the intrinsic VCM governed by V_s_. Aligned with the recent progress in defect engineering, this transition leverages the controllable generation and migration of V_S_ as the primary switching medium. As demonstrated in previous studies on transition-metal dichalcogenides, the precise regulation of atomic vacancies is a pivotal strategy to tailor local electronic structures [33,34]. Under the driving electric field, the localized generation and alignment of these intrinsic vacancies form finer and highly controllable conductive channels, thereby suppressing the lateral diffusion typically observed in overgrown metallic filaments. Upon applying a reverse voltage, these fine vacancy channels rupture more thoroughly through ion recombination, leading to the hydrophobic-side memristor’s improved uniformity, larger switching window, and enhanced endurance.

### 3.4. Multimodal Neuromorphic Characteristics of Janus-MoS_2_-Based Electronic Synapse

**Figure 4:**
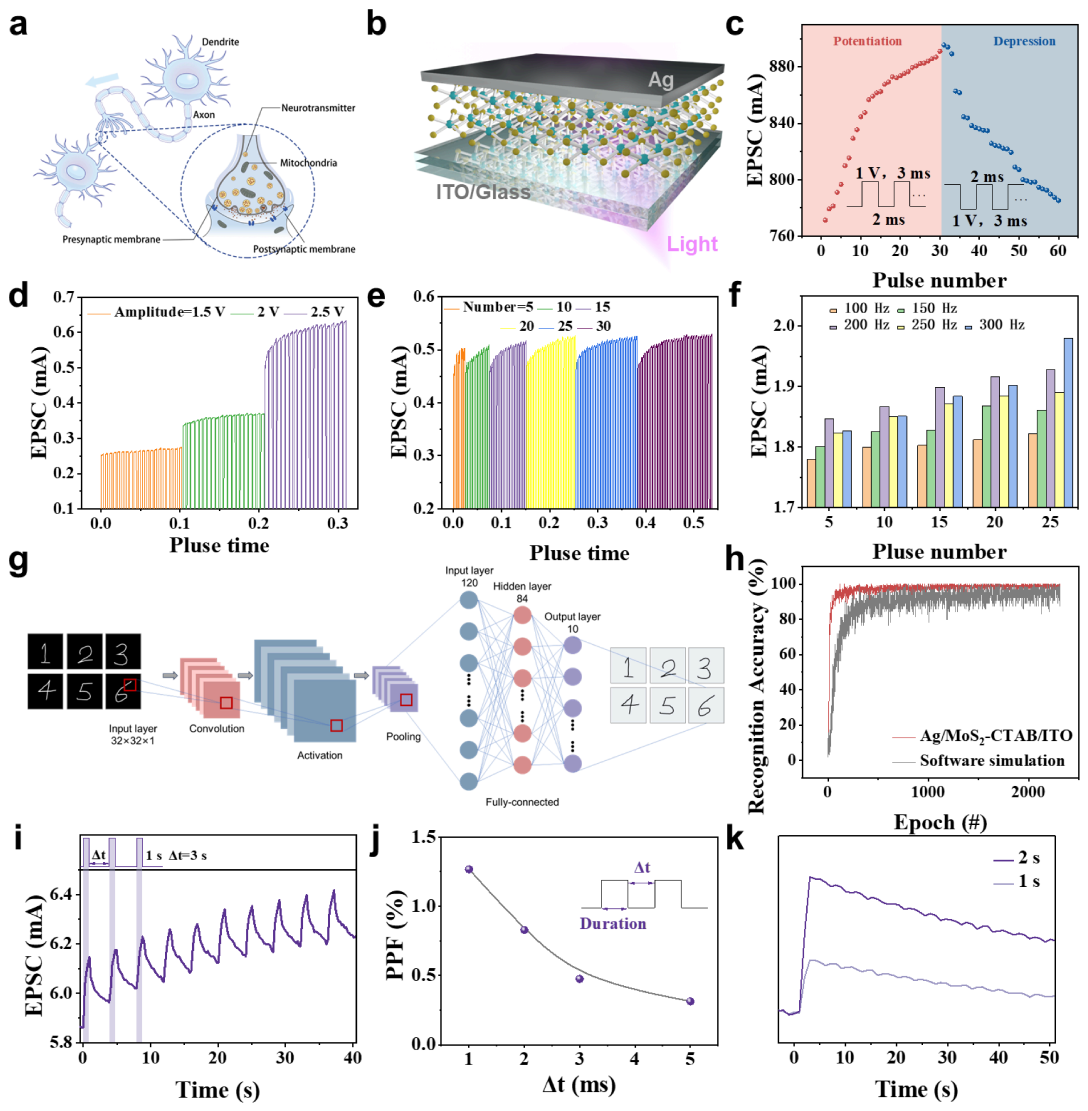
Electronic synaptic characteristics and 405 nm light response of MoS_2_ film-based devices. (**a**) Structure of a biological synapse and the process of action potential transmission from the presynaptic to the postsynaptic membrane. (**b**) Schematic diagram of the light-response mechanism based on the MoS_2_ film. (**c**) Continuous conductance modulation of the memristor based on the hydrophilic side of MoS_2_. (**d**–**f**) Ag/MoS_2_/ITO device emulating synaptic behaviors including SADP, SNDP, SRDP. (**g**) Schematic illustration of a convolutional neural network for image recognition (MNIST dataset). The red square represents the convolution kernel, which slides over the input image to extract features. The red and blue circles represent the artificial neurons in the hidden and output layers, respectively, which are fully connected. (**h**) Evolution of recognition accuracy during training (Epoch #) based on the Ag/Janus-structured MoS_2_/ITO memristor on the hydrophobic side of the MoS_2_ film (red curve), compared with the software simulation (gray curve). (**i**) Current response to 405 nm light pulses with a duration of 1 s and an interval of 1 s. (**j**) PPF behavior of the device under 1 s light pulses. (**k**) Comparison of current changes after 1 s and 2 s light illumination, demonstrating the nonlinear enhancement of the postsynaptic current with increased illumination duration.

Figure 4a shows the schematic of a biological synapse structure, illustrating information transmission in the nervous system: when an action potential reaches the presynaptic membrane, neurotransmitters are released and cross the synaptic cleft. They bind to receptors on the postsynaptic membrane, inducing changes in the postsynaptic potential, thereby achieving signal transmission and modulation from pre- to postsynaptic neurons. To explore the potential of Janus-structured MoS_2_-based memristors for neuromorphic applications, we investigated the biomimetic characteristics and application potential of Janus-structured MoS_2_ memristors as artificial synaptic devices through a series of pulse tests. By applying continuous bipolar pulse trains, we successfully simulated LTP and LTD behaviors (Figure 4c), which serve as the physical basis for synaptic weight quantization in neural networks. Various typical forms of synaptic plasticity, including LTP and Short-Term Plasticity (STP), were emulated using different voltage pulse waveforms. In particular, Spike-Amplitude-Dependent Plasticity (SADP), Spike-Number-Dependent Plasticity (SNDP), and Spike-Rate-Dependent Plasticity (SRDP) within LTP were simulated by varying pulse amplitude, number, and frequency, respectively (Figure 4d–f). To further verify the role of the relative timing order of pre- and postsynaptic neuronal spikes in synaptic weight modulation, Spike-Timing-Dependent Plasticity (STDP) was studied by applying paired pre- and postsynaptic pulses with specific temporal delays (Figure S19). Paired-Pulse Facilitation (PPF) and Paired-Pulse Depression (PPD) within STP were simulated by applying two consecutive pulses with the same voltage but different inter-pulse intervals (Figure S20). The extracted relaxation time constants are t_1_ = 3.64 ms and 1.82 ms for the excitatory and inhibitory processes, respectively. The distinct difference between these time constants confirms the asymmetric synaptic dynamics of our device, which effectively mimics the complex temporal processing capabilities of biological synapses and provides a robust physical basis for its application in neuromorphic hardware acceleration. For devices with stabilized conductive filaments, applying 100 consecutive bipolar pulse trains resulted in sudden current increases and sharp drops (Figure S21). This non-linear switching characteristic reflects the operational mechanism involving the formation and rupture of conductive pathways based on silver ions or V_s_ under the application of an electric field.

To further evaluate the potential of Janus-structured MoS_2_-based synapses for neuromorphic computing, a Convolutional Neural Network (CNN) model was constructed based on experimental device data. A classic handwritten digit recognition simulation was performed using the architecture shown in Figure 4g, consisting of input, convolutional, pooling, and fully connected layers. After training the CNN based on the backpropagation algorithm on a dataset of 60,000 handwritten digit images (the detailed network framework and simulation parameters are provided in Note S1), its recognition accuracy was tested on 10,000 images. The recognition rate rapidly increased with the number of iterations and showed good convergence. After a certain number of training epochs, the network based on Janus-structured MoS_2_ memristors achieved a higher recognition rate compared to traditional software-driven floating-point networks. The device not only enabled high-precision weight quantization but also required fewer iterations to reach high accuracy, demonstrating the significant potential of Janus-structured MoS_2_ memristors for constructing low-power, high-efficiency neuromorphic hardware systems.

Beyond electrical stimulation, previous UV characterization confirmed that Janus-structured MoS_2_ films exhibit significant absorption from the UV to visible light range, providing the physical basis for light-induced conductance modulation. The RS characteristics of Janus-structured MoS_2_ memristors under 405 nm laser illumination were explored to reveal their potential for photo-electro synergistic regulation. A near-UV (405 nm) light beam was vertically irradiated from below the device through the transparent ITO glass substrate (Figure 4b), while a constant 0.1 V bias was applied to monitor the current response. The device was subjected to fixed 1 s light pulses (Figure 4i and Figure S22) with systematically varied inter-pulse intervals (1 s, 2 s, 3 s, and 5 s). Upon illumination, the device current instantly increased and then slowly decayed when the light was turned off. Under continuous light pulses, the current did not fully return to its initial state during the intervals but exhibited a distinct stepwise increment and a stepwise accumulation trend. This suggests that photogenerated carriers have specific relaxation lifetimes; the residual carriers from the previous pulse superimpose with new ones generated by the subsequent pulse, leading to a cumulative enhancement effect. To investigate short-term memory retention and relaxation dynamics, the single-pulse duration was set to 0.2 s, and the interval between pulses was systematically varied (Figure S23). The PPF index, defined as the ratio of current gains induced by two consecutive light pulses, was calculated for both 0.2 s and 1 s pulse durations (Figure 4j and Figure S24). The PPF index monotonically decreased with increasing inter-pulse interval. At short intervals, the device showed maximum facilitation gain, analogous to the strong capture of transient high-frequency signals in biological vision. As the interval increased, the PPF index gradually decreased toward zero, indicating that photogenerated carriers had more time to recombine, diffuse, or be trapped at deep-level defects, allowing the device to recover closer to its initial state. This tunable PPF behavior simulates “short-term visual adaptation” in biological visual pathways. The effect of single-pulse duration on synaptic weight change was studied by applying 1 s and 2 s light pulses of the same wavelength and power to the same device. The postsynaptic current exhibited a significant, non-linear multiplication effect with increasing illumination time, rather than a simple linear increase. This indicates that prolonged illumination continuously generates new electron-hole pairs, while previously generated carriers accumulate before recombination, leading to accelerated current enhancement due to increased charge density over time. This photo-electro synergistic mechanism enables Janus-structured MoS_2_ memristors to function not only as electrical memory units but also as photosensitive synapses, integrating the perception, processing, and storage of external optical stimuli, thereby laying a foundation for the development of brain-like visual perception systems.

## 4. Conclusions

In this study, 2H-phase MoS_2_ nanosheet dispersions were prepared via an electrochemical method using CTAB as the intercalation molecule, and large-area uniform Janus-structured MoS_2_ films were obtained by transferring the nanosheets at an oil–water interface through a self-assembly technique. We further constructed Janus-structured MoS_2_-based memristors, which exhibited excellent non-volatile memory characteristics across both interface configurations, including a high cycling endurance (>400 cycles) with highly stable switching parameter distributions, and an ultralong data retention (>40,000 s). Mechanistic exploration reveals a distinct divergence in their underlying switching principles: while the hydrophilic side is governed by the ECM mechanism, the dense organic modification layer on the hydrophobic side acts as a crucial ion buffer and barrier to effectively suppress the disordered migration of silver ions, thereby shifting its dominant resistive switching mechanism to a highly controllable, intrinsic VCM dominated by V_s_. Meanwhile, the Ag/Janus-structured MoS_2_/ITO structure successfully emulated various synaptic learning rules induced by electrical pulses, including STP (e.g., PPF and PPD), LTP (e.g., SADP, SNDP, SRDP, and STDP), and LTD. The devices were also applied to image-recognition tasks, showing promising recognition accuracy. Furthermore, by introducing 405 nm light pulses as a co-regulatory stimulus, photo-synaptic functionality was realized in the same device, demonstrating the potential for constructing integrated sensing–storage–computing systems. Overall, this work presents an effective strategy for tuning the performance of two-dimensional material-based memristors through surface modification and for extending their optoelectronic neuromorphic functionalities.

## Data Availability

The original contributions presented in this study are included in the article/Supplementary Materials. Further inquiries can be directed to the corresponding authors.

## Supplementary Materials

The following supporting information can be downloaded at https://www.mdpi.com/article/10.3390/nano16150959/s1. Figure S1: Electrochemical cell during electrochemical intercalation of MoS_2_; Figure S2: Characterization of natural bulk MoS_2_, sonicated MoS_2_ solution without intercalation, and MoS_2_ nanosheet dispersion; Figure S3: TEM characterization of (a,b) bulk MoS_2_ natural crystals and (c,d) the MoS_2_ nanosheet dispersion; Figure S4: SEM characterization of (a,b) bulk MoS_2_ natural crystals and (c,d) the MoS_2_ nanosheet dispersion; Figure S5: AFM image of the MoS_2_ nanosheet dispersion; Figure S6: Experimental parameters for the preparation of MoS_2_ nanosheet dispersions via electrochemical intercalation; Figure S7: Raman spectra of MoS_2_ nanosheet dispersions prepared under different conditions; Figure S8: AFM morphological characterization of MoS_2_ nanosheet dispersions under different preparation conditions; Figure S9: Transfer diagram of MoS_2_ thin films at the oil-water interface; Figure S10: EDS mappings of the hydrophilic and hydrophobic surfaces of the MoS_2_ film; Figure S11: XPS survey spectra of (a) the hydrophilic surface and (b) the hydrophobic surface of the MoS_2_ film, and (c) the atomic concentration table obtained from XPS analysis; Figure S12: High-resolution XPS spectra of (a) C 1s, (b) N 1s, (c) Mo 3d, and (d) S 2p for the hydrophilic surface of the MoS_2_ film; and (e) C 1s, (f) N 1s, (g) Mo 3d, and (h) Si 2p for the hydrophobic surface of the MoS_2_ film; Figure S13: AFM height images and corresponding thickness profiles of the self-assembled Janus MoS_2_ films; Figure S14: Comparison of UV-Vis spectra of the hydrophilic surface and the hydrophobic surface of the MoS_2_ film; Figure S15: Optical images of silver electrodes on (a) the hydrophilic surface and (b) the hydrophobic surface of the MoS_2_ film; Figure S16: Frequency distribution histograms of set and reset voltages for 420 I–V cycles of the Ag/Janus-structured MoS_2_/ITO memristor; Figure S17: Device-to-device resistance distributions and yield evaluation based on 50 independent memristors for (a) hydrophilic side and (b) hydrophobic side configurations, and (c) ON/OFF ratio; Figure S18: Benchmarking of memory performance parameters between this work and available MoS_2_ literature; Figure S19: STDP behaviors of the biological synapse simulated by the MoS_2_-based memristor; Figure S20: PPF (a) and PPD (b) behaviors of the biological synapse simulated by the MoS_2_-based memristor; Figure S21: Current variations of the device with a formed conductive filament under 100 consecutive (a) 1 V pulses and (b) −1 V pulses; current variations of the device without a formed conductive filament under 100 consecutive (c) 1 V pulses and (d) 1.5 V pulses; Note S1: Framework and Details of the Neuromorphic MNIST Simulation; Figure S22: Current response curves of the MoS_2_-based memristor under 1 s light pulses with different time intervals; Figure S23: Current response curves of the MoS_2_-based memristor under 0.2 s light pulses with different time intervals; Figure S24: PPF index as a function of interval time between 0.2 s light pulses (a) and 1 s light pulses (b) for the MoS_2_-based memristor. References [41,42,43,44,45,46] are cited in the Supplementary Materials.

## Abbreviations

The following abbreviations are used in this manuscript:

2D	Two-dimensional
CTAB	Cetyltrimethylammonium bromide
TMDs	Transition metal dichalcogenides
MoS_2_	Molybdenum disulfide
STP	Short-term plasticity
LTP	Long-term plasticity
CVD	Chemical vapor deposition
DMF	N,N-dimethylformamide
PVP	Polyvinylpyrrolidone
O/W	Oil/water
TEM	Transmission electron microscopy
SEM	Scanning electron microscopy
XPS	X-ray photoelectron spectroscopy
ATR-FTIR	Attenuated total reflection Fourier transform infrared spectroscopy
HRS	High-resistance state
LRS	Low-resistance state
SADP	Spike-Amplitude-Dependent Plasticity
SNDP	Spike-Number-Dependent Plasticity
SRDP	Spike-Rate-Dependent Plasticity
STDP	Spike-Timing-Dependent Plasticity
PPF	Paired-Pulse Facilitation
PPD	Paired-Pulse Depression
CNN	Convolutional Neural Network
