# Supplementary material for: Interfacial Engineering of MoS2 Thin Films for Wettability-Dependent Resistive Switching and Neuromorphic Behaviors
Source: Nanomaterials (Basel). 2026 Aug 4;16(15):959. doi: 10.3390/nano16150959 (PMC13468638; doi:10.3390/nano16150959)
Supplement: Supplementary file 1 [file nanomaterials-16-00959-s001.zip › nanomaterials-4383835-supplementary.pdf]

Supporting Information

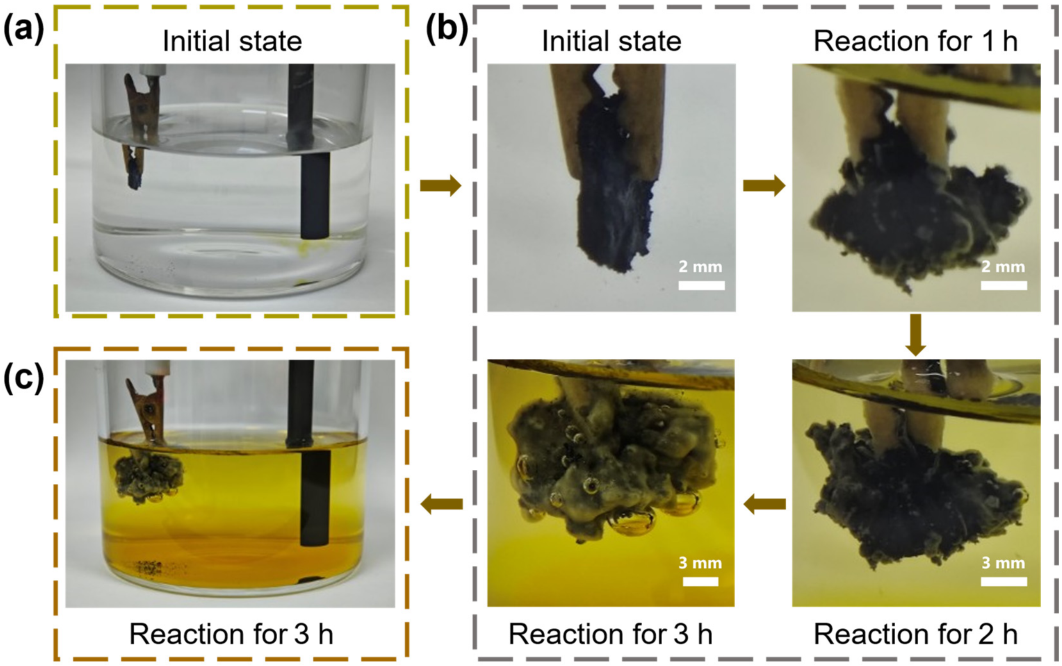

Figure S1. Electrochemical cell during electrochemical intercalation of MoS_2. (a) Electrochemical cell at the start of applying 8 V (initial state) and (b) after 3 hours of MoS_2 intercalation; (c) Expansion states of MoS_2 crystals after different intercalation hours.

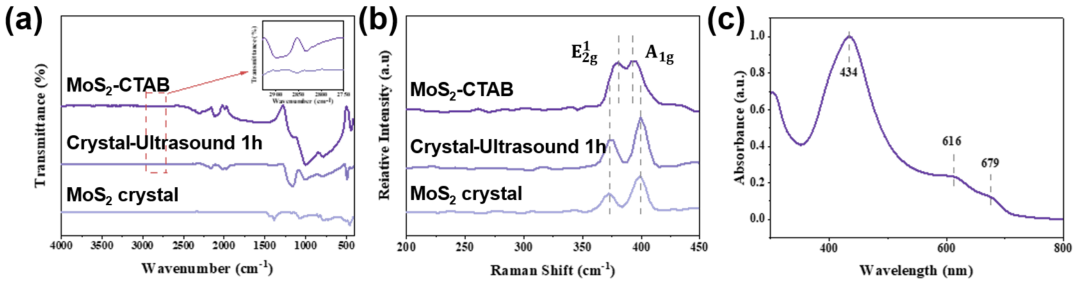

Figure S2. Characterization of natural bulk MoS_2, sonicated MoS_2 solution without intercalation, and MoS_2 nanosheet dispersion: (a) ATR-FTIR spectra, (b) Raman spectra, and (c) UV-Vis absorption spectrum of the MoS_2 nanosheet dispersion.

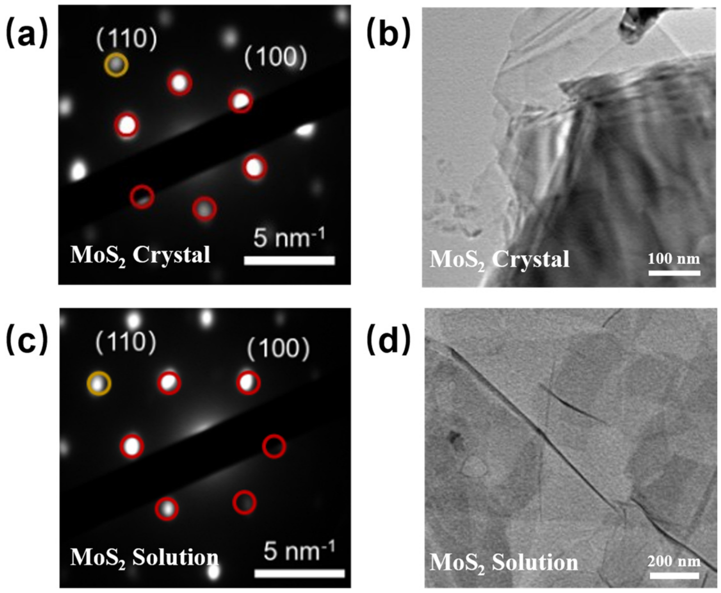

Figure S3. TEM characterization of (a–b) bulk MoS_2 natural crystals and (c–d) the MoS_2 nanosheet dispersion.

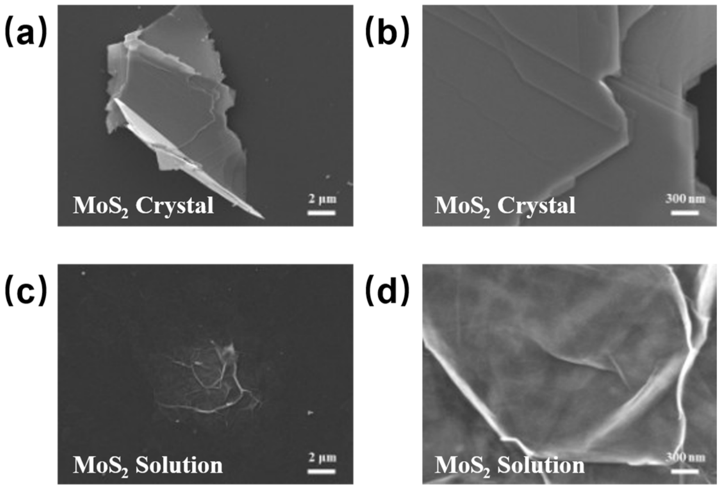

Figure S4. SEM characterization of (a–b) bulk MoS_2 natural crystals and (c–d) the MoS_2 nanosheet dispersion.

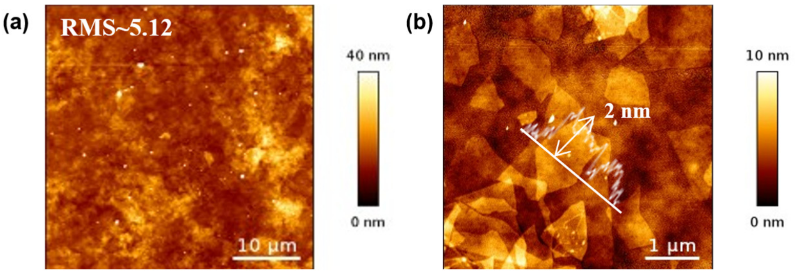

Figure S5. AFM image of the MoS_2 nanosheet dispersion.

<table>
<tr><th>Interstitial molecules</th><th>Electrolyte concentration</th><th>Reaction time</th><th>Ultrasound time</th><th>Centrifugal speed</th></tr>
<tr><td rowspan="7">CTAB</td><td rowspan="6">5 mg/mL</td><td>1 h</td><td>1 h</td><td>3000 rpm</td></tr>
<tr><td rowspan="6">3 h</td><td rowspan="4">1 h</td><td>3000 rpm</td></tr>
<tr><td>2000 rpm</td></tr>
<tr><td>1500 rpm</td></tr>
<tr><td>1000rpm</td></tr>
<tr><td>0.5 h</td><td>3000rpm</td></tr>
<tr><td>8 mg/mL</td><td>1 h</td><td>3000 rpm</td></tr>
</table>

Figure S6. Experimental parameters for the preparation of MoS_2 nanosheet dispersions via electrochemical intercalation.

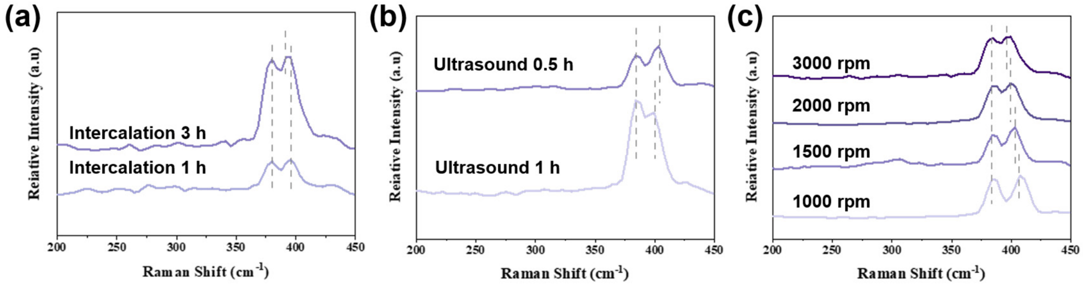

Figure S7. Raman spectra of MoS_2 nanosheet dispersions prepared under different conditions: (a) varying intercalation durations, (b) varying ultrasonication durations, and (c) varying centrifugation speeds.

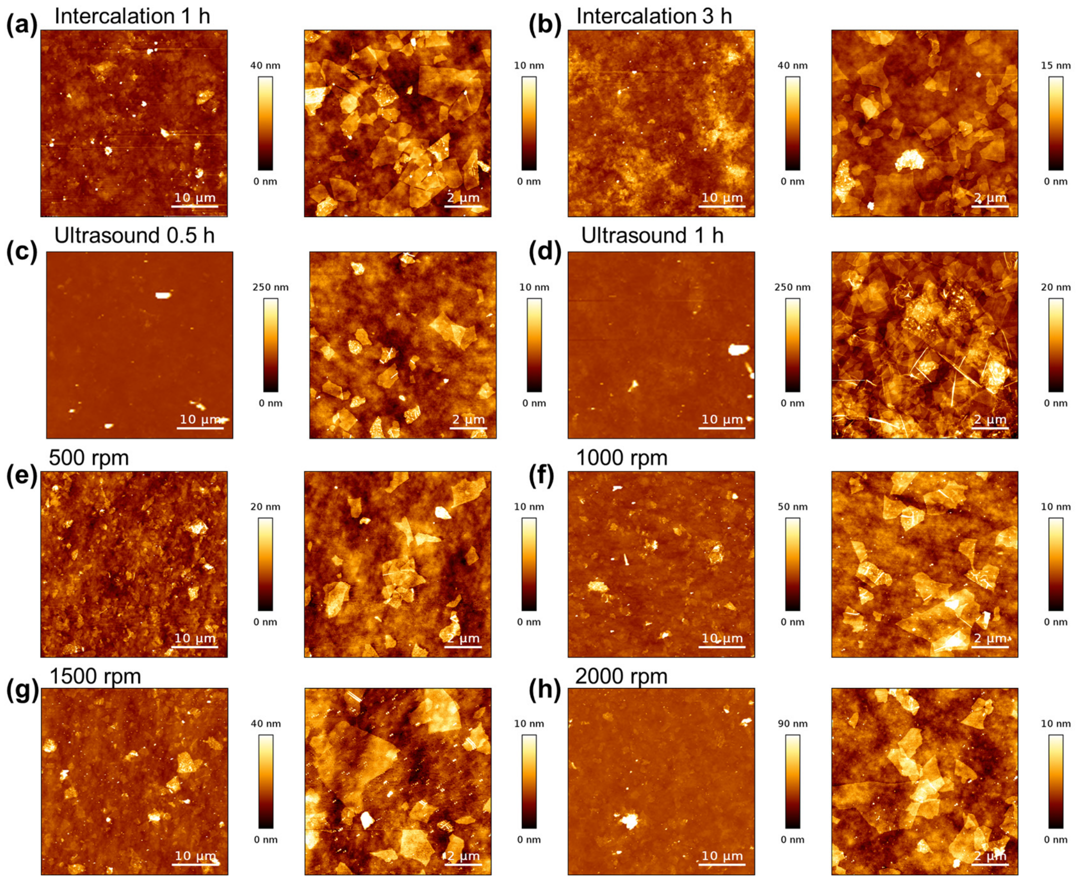

Figure S8. AFM morphological characterization of MoS_2 nanosheet dispersions under different preparation conditions: (a-b) Comparison between 1 h and (c-d) 3 h of electrochemical intercalation; (e-f) Size distribution of nanosheets exfoliated by sonication for 0.5 h and 1 h; (g-j) Morphological evolution at different centrifugation speeds (500, 1000, 1500, and 2000 rpm).

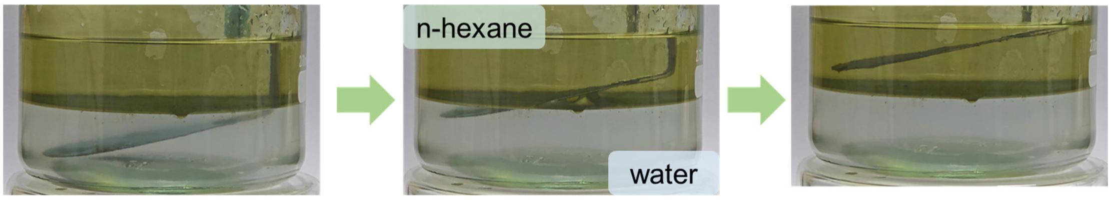

Figure S9. Transfer diagram of MoS_2 thin films at the oil-water interface.

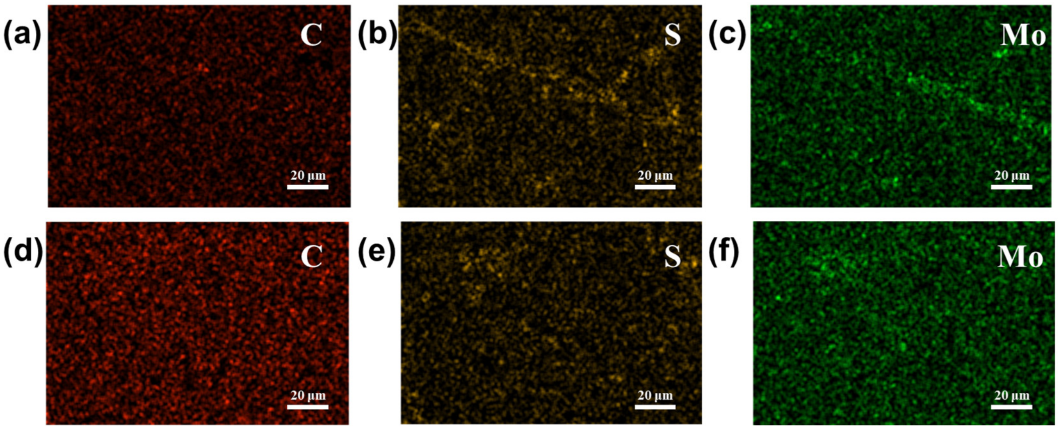

Figure S10. EDS mappings of the hydrophilic and hydrophobic surfaces of the MoS_2 film. Elemental distributions of (a) C, (b) S, and (c) Mo on the hydrophilic surface; and (d) C, (e) S, and (f) Mo on the hydrophobic surface.

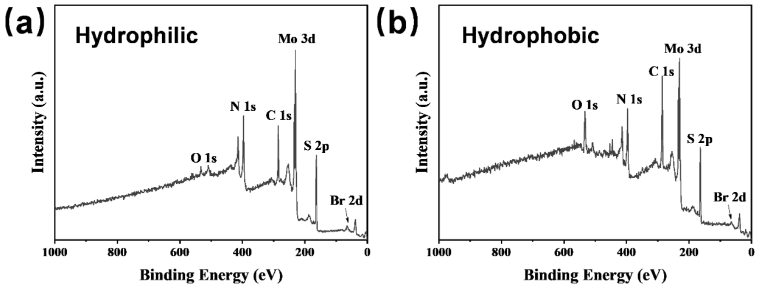

(c)

Element	Hydrophilic (At. %)	Hydrophobic (At. %)
C 1s	31.61	48.76
Mo 3d	12.37	9.40
N 1s	34.02	25.44
S 2p	21.82	16.12
Br 3d	0.18	0.28

Figure S11. XPS survey spectra of (a) the hydrophilic surface and (b) the hydrophobic surface of the MoS_2 film, and (c) the atomic concentration table obtained from XPS analysis.

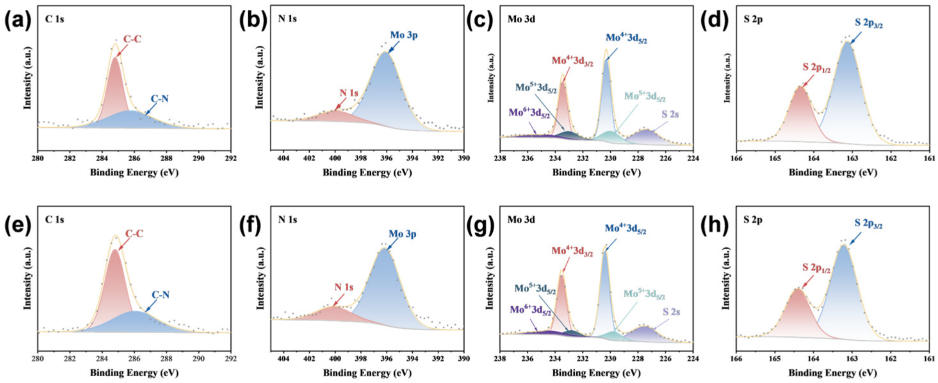

Figure S12. High-resolution XPS spectra of (a) C 1s, (b) N 1s, (c) Mo 3d, and (d) S 2p for the hydrophilic surface of the MoS_2 film; and (e) C 1s, (f) N 1s, (g) Mo 3d, and (h) Si 2p for the hydrophobic surface of the MoS_2 film.

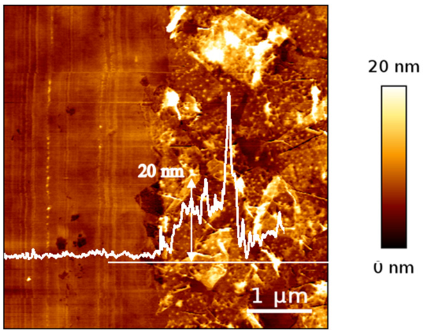

Figure S13. AFM height images and corresponding thickness profiles of the self-assembled Janus MoS_2 films.

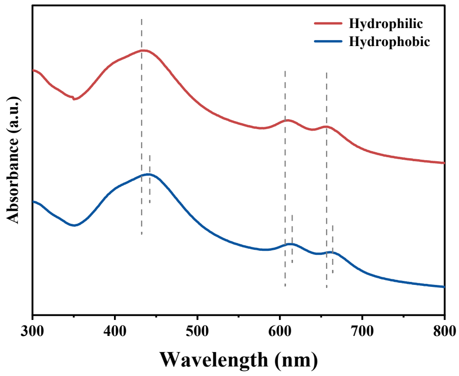

Figure S14. Comparison of UV-Vis spectra of the hydrophilic surface and the hydrophobic surface of the MoS_2 film. The vertical dashed lines indicate the positions of the characteristic absorption peaks

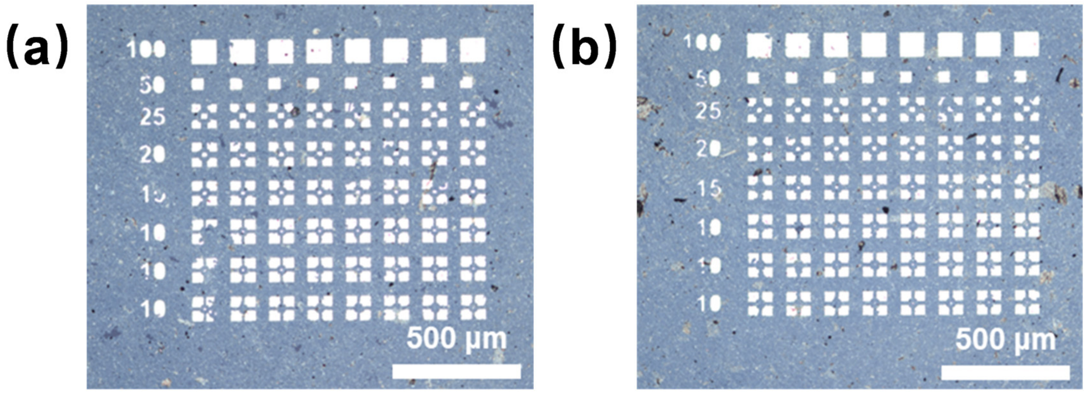

Figure S15. Optical images of silver electrodes on (a) the hydrophilic surface and (b) the hydrophobic surface of the MoS_2 film. The device sizes ranges from 10 μm to 100 μm.

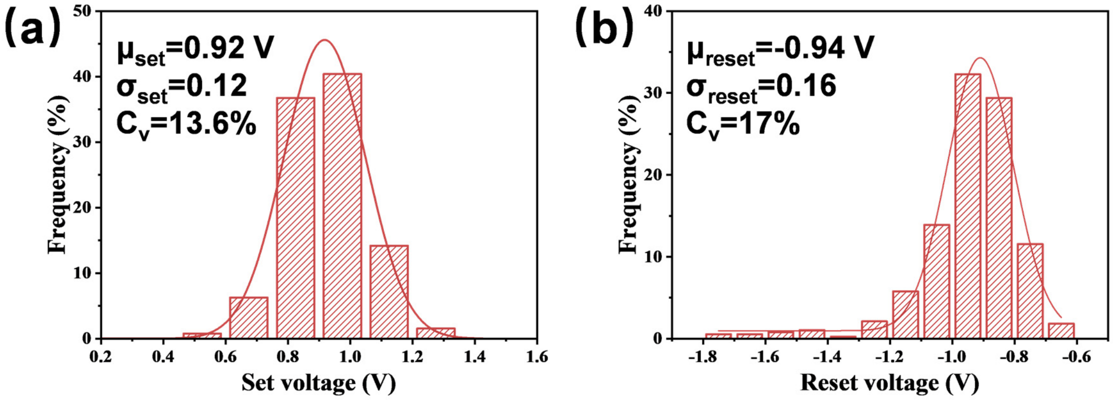

Figure S16. Frequency distribution histograms of set and reset voltages for 420 I-V cycles of the Ag/Janus-structured MoS_2/ITO memristor.

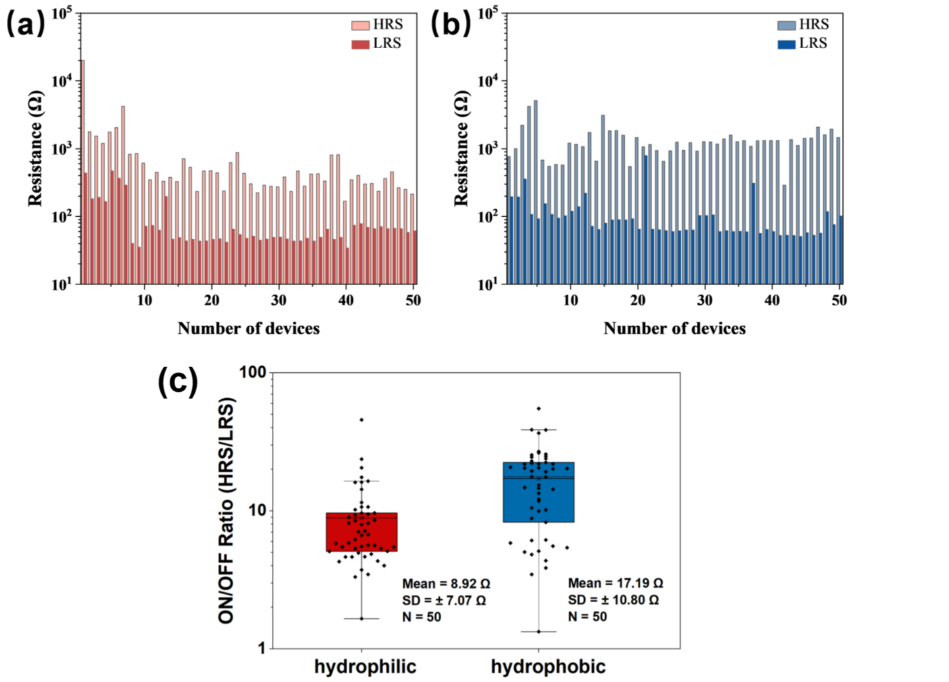

Figure S17. Device-to-device resistance distributions and variability evaluation based on 50 independent memristors for (a) hydrophilic side, (b) hydrophobic side configurations, and (c) ON/OFF ratio.

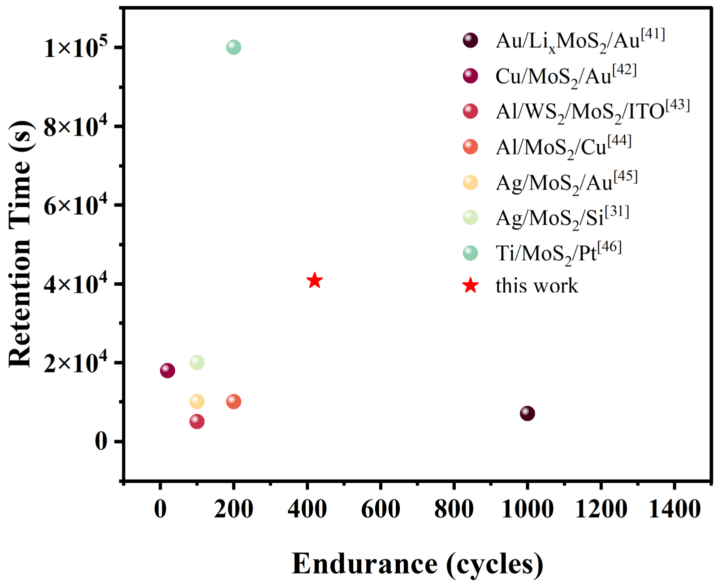

Figure S18. Benchmark comparison of endurance and retention performance for MoS_2-based memristors reported in the literature.

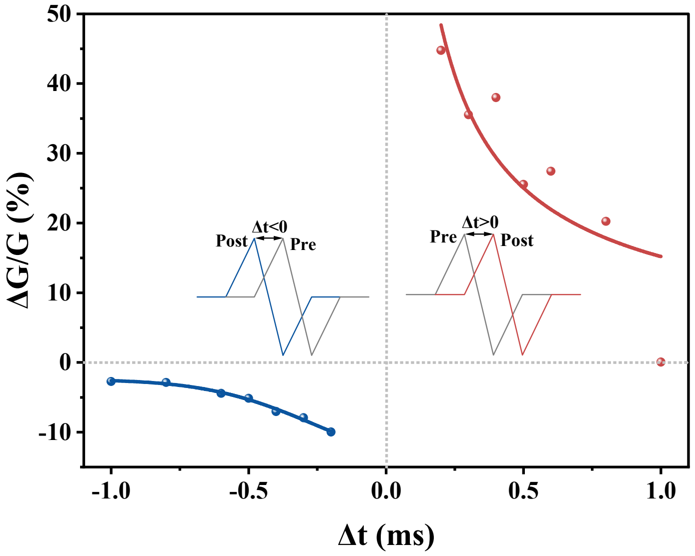

Figure S19. STDP behaviors of the biological synapse simulated by the MoS_2-based memristor.

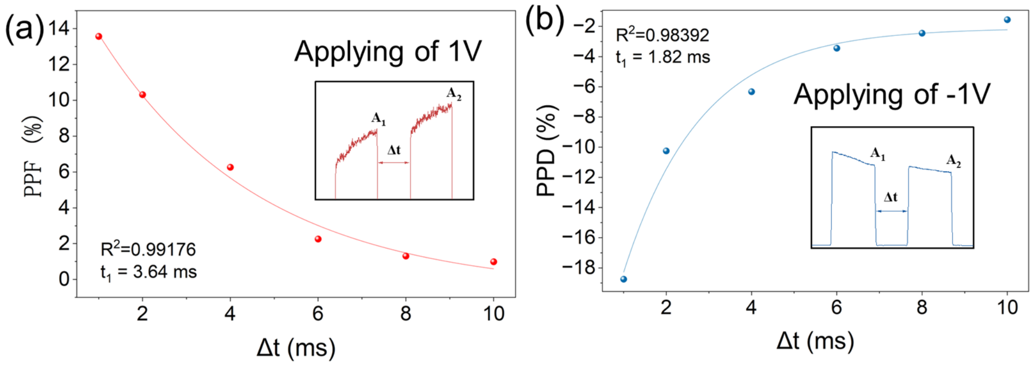

Figure S20. PPF (a) and PPD (b) behaviors of the biological synapse simulated by the MoS_2-based memristor.

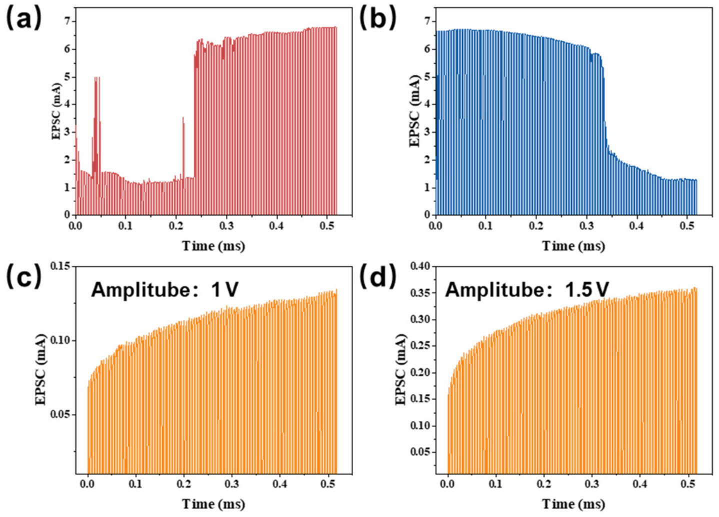

Figure S21. Current variations of the device with a formed conductive filament under 100 consecutive (a) 1 V pulses and (b) -1 V pulses; current variations of the device without a formed conductive filament under 100 consecutive (c) 1 V pulses and (d) 1.5 V pulses.

Note S1. Framework and Details of the Neuromorphic MNIST Simulation

To quantitatively evaluate the hardware-level learning and recognition capability of the Janus-structured MoS_2-based memristors, an artificial neural network (ANN) simulation was executed based on an empirical behavioral mapping model. The continuous analog conductance tuning kinetics (Long-Term Potentiation and Long-Term Depression) obtained from our hardware characterizations were mathematically normalized and extracted, serving as the realistic boundary parameters for synaptic weight modulation (W).

1. Network Architecture and Implementation:

Input Layer: Consists of 784 input nodes, where each node represents a single pixel from the standard 28×28 pixels MNIST handwritten digit images. The grayscale values of the pixels are normalized into corresponding input vectors.

Hidden Layer: Employs 100 hidden neurons configured with a standard activation function to extract and map complex structural features of the handwritten digits.

Output Layer: Features 10 designated output nodes representing the classification results for digits from 0 to 9. The final classification is determined using the "winner-take-all" scheme based on the maximum output intensity.

2. Simulation Dynamics and Convergence:

During the training process, the standard dataset containing 60,000 training samples was processed utilizing the error backpropagation algorithm. The error generated in the output layer is propagated backward to dynamically adjust the weight matrix governed by the realistic device conductance switching behaviors.

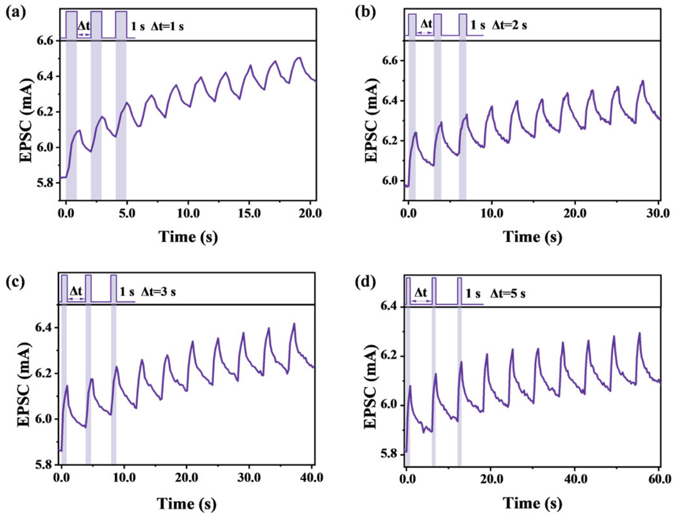

Figure S22. Current response curves of the MoS_2-based memristor under 1 s light pulses with different time intervals.

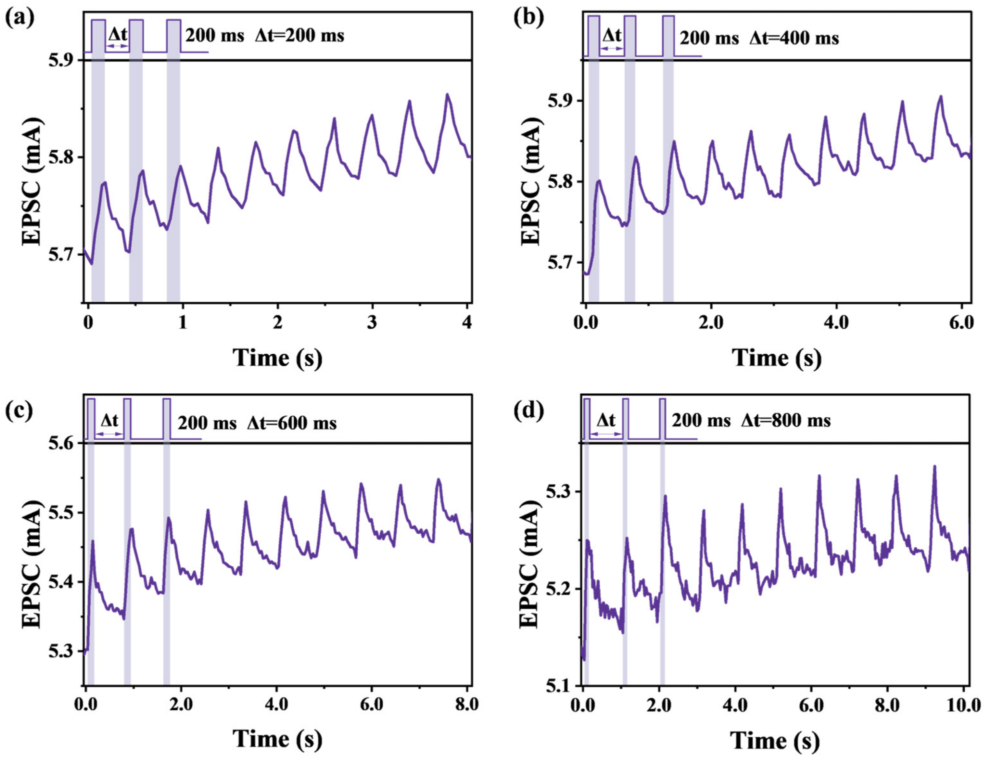

Figure S23. Current response curves of the MoS_2-based memristor under 0.2 s light pulses with different time intervals.

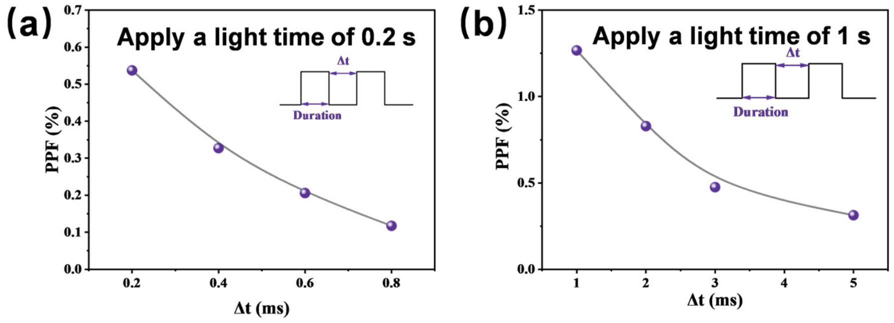

Figure S24. PPF index as a function of interval time between 0.2 s light pulses (a) and 1 s light pulses (b) for the MoS_2-based memristor.

1. Zhu, X.; Li, D.; Liang, X.; Lu, W.D. Ionic modulation and ionic coupling effects in MoS2 devices for neuromorphic computing. *Nat. Mater.* **2018**, *18*, 141–148, https://doi.org/10.1038/s41563-018-0248-5.
2. Xu, R.; Jang, H.; Lee, M.-H.; Amanov, D.; Cho, Y.; Kim, H.; Park, S.; Shin, H.-J.; Ham, D. Vertical MoS_2 Double-Layer Memristor with Electrochemical Metallization as an Atomic-Scale Synapse with Switching Thresholds Approaching 100 mV. *Nano Lett.* **2019**, *19*, 2411–2417, https://doi.org/10.1021/acs.nanolett.8b05140.
3. Zhang, W.; Gao, H.; Deng, C.; Lv, T.; Hu, S.; Wu, H.; Xue, S.; Tao, Y.; Deng, L.; Xiong, W. An ultrathin memristor based on a two-dimensional WS_2/MoS_2 heterojunction. *Nanoscale* **2021**, *13*, 11497–11504, https://doi.org/10.1039/d1nr01683k.
4. Chen, J.; Xu, J.; Chen, J.; Gao, L.; Yang, C.; Guo, T.; Zhao, Y.; Xiao, Y.; Wang, J.; Li, Y. High-performance memristor based on MoS2 for reliable biological synapse emulation. *Mater. Today Commun.* **2022**, *32*, https://doi.org/10.1016/j.mtcomm.2022.103957.
5. Zhao, X.; Fan, Z.; Xu, H.; Wang, Z.; Xu, J.; Ma, J.; Liu, Y. Reversible alternation between bipolar and unipolar resistive switching in Ag/MoS $_2$ /Au structure for multilevel flexible memory. *J. Mater. Chem. C* **2018**, *6*, 7195–7200, https://doi.org/10.1039/c8tc01844h.
6. Tang, B.; Veluri, H.; Li, Y.; Yu, Z.G.; Waqar, M.; Leong, J.F.; Sivan, M.; Zamburg, E.; Zhang, Y.-W.; Wang, J.; et al. Wafer-scale solution-processed 2D material analog resistive memory array for memory-based computing. *Nat. Commun.* **2022**, *13*, 1–9, https://doi.org/10.1038/s41467-022-30519-w.
